# Consequences of Sensory Modality for Perspective-Taking: Comparing Visual, Olfactory and Gustatory Perception

**DOI:** 10.3389/fpsyg.2021.701486

**Published:** 2021-08-16

**Authors:** Elsi Kaiser

**Affiliations:** Department of Linguistics, University of Southern California, Los Angeles, CA, United States

**Keywords:** perspective-taking, subjective adjectives, predicates of personal taste, senses, psycholinguistics

## Abstract

Perspective-taking is fundamental for language comprehension, including the interpretation of subjective adjectives (e.g., *fun, tasty*, and *amazing*). To understand these adjectives, one needs to know whose opinion is being conveyed—in other words, who is the attitude-holder or perspectival center. Although the perspective-sensitivity of subjective adjectives has received considerable attention in prior work in formal semantics, potential effects of sensory modality (e.g., sight, taste, and smell) on the process of attitude-holder identification have not been systematically investigated. This paper reports a series of studies testing whether interpretation of subjective adjectives depends on whether they refer to the visual, olfactory (smell) vs. gustatory (taste) domains. The results provide evidence that sensory modality has a significant impact on the process of identifying the attitude-holder. This outcome suggests that perspective-sensitivity is highly context-dependent, and the observed modality effects align well with the biological and social properties of sight, taste, and smell.

## Introduction

Subjective opinions are fundamental to human cognition and perception (e.g., Markus and Zajonc, 1985; Jarvis and Petty, 1996), and language contains a wide range of subjective expressions, including a class of adjectives known as predicates of personal taste (PPTs), such as *fun, tasty, disgusting, amazing*, that reflect opinions. Intuitively, these subjective adjectives differ from objective adjectives, such as *wooden, organic* or *Finnish*. If two people disagree about an objective statement such as “This apple is organic,” one of them must be in the wrong. However, people can disagree about a subjective statement such “This apple is tasty” without anyone being in the wrong [e.g., Koelbel (2004) on faultless disagreement]: one person might find an apple to be tasty while another finds the same apple too sour. As the old adage goes, there's no arguing about taste. Thus, to fully understand subjective adjectives, a comprehender needs to know whose opinion/attitude is being conveyed. Understanding that a certain food is tasty to a cat is different from certain food is tasty to a human, for example. In theoretical analyses, PPTs are often described as making reference to a judge, attitude-holder or evaluator [e.g., Lasersohn (2005, 2007), Potts (2007), Stephenson (2007), Patel-Grosz (2012), but see also Pearson (2013), Coppock (2018)]. Thus, intuitively, a fundamental aspect of successfully comprehending a PPT involves identification of the intended attitude-holder^1^.

Although PPTs have received considerable attention in theoretical semantics and philosophy, to the best of my knowledge, semantically-oriented theories of PPTs do not explicitly make systematic distinctions based on sensory modality—in other words, whether the evidence on which the subjective opinion is based stems from visual, auditory, gustatory, olfactory, or tactile experience. Broadly speaking, under these accounts, a sentence such as “It was disgusting” is analyzed the same way semantically regardless of whether it refers to the taste, smell or visual appearance of a pizza slice, for example. These accounts are not incompatible with differences between sensory modalities, they simply do not make explicit predictions about them. This leaves open the (more pragmatic) question of whether comprehenders' interpretations of subjective adjectives—in particular, who is the intended attitude-holder—differ based on sensory modality. If yes, this can inform our understanding of the interplay between non-linguistic real-world knowledge and the interpretation of linguistic elements. Thus, one of the aims of the present paper is to systematically investigate whether modality matters in how language users identify the intended attitude-holder of PPTs.

Moreover, although the notion of perspective-taking has received extensive attention in psycholinguistic research [see e.g., Brown-Schmidt and Heller (2018) for a recent review], perspective-taking of the type involved with subjective predicates has received less attention. Most prior psycholinguistic work has focused on objective situations—contexts where one person has access to factually-correct information that the other person cannot access (see e.g., Keysar et al., 2000; Hanna et al., 2003; Heller et al., 2008). In the case of subjective opinions, everyone can have access to the same objective information but still arrived at different subjective opinions [and can all be equally “in the right,” e.g., Koelbel (2004)]. The present paper aims to inform our understanding of perspective-taking in subjective contexts.

This paper reports three experiments testing if identification of the attitude-holder of subjective adjectives (specifically PPTs) in English is influenced by (i) whether the adjective is presented in a modality-neutral way or associated with a sensory modality, and (ii) whether different sensory modalities differ in how they impact the process of attitude-holder identification. Before taking a closer look at the hypotheses, let us first consider why one might expect sensory modalities to differ with respect to attitude-holder identification. With this goal in mind, the next section reviews work on the biological properties of different senses, their perceived subjectivity, and the ease of accessing sensory stimuli in different modalities.

## Sensory Modalities

### Vision, Taste, and Smell

It is well-known that the five traditional senses (sight/vision, hearing/audition, taste/gustation, touch/feel and smell/olfaction) are fundamentally different, not only in their biological underpinnings but also in terms of their (i) perceived level of subjectivity and (ii) spatiotemporal properties and associated differences in ease of perceptual access. The subsequent sections review the relevant properties of vision, taste and smell, the senses investigated in this paper^2^. We hope that this paper can serve as a foundation for future work on other sensory modalities.

#### Perceived Subjectivity

Vision is commonly viewed as the dominant sense in most human cultures and languages [e.g., San Roque et al. (2015), but contra Aikhenvald and Storch (2013)]. Biologically, vision is a highly specialized sense in humans, and by some estimates, up to 50% of the cortex is involved in visual functions (Palmer, 1999). Research on sensory dominance effects suggests that visual input tends to dominate over auditory input if the two are in conflict (Colavita, 1974; Sinnett et al., 2007; Spence, 2009)—humans may have a biologically hardwired preference to rely on vision [but see Aglioti and Pazzaglia (2010)].

As regards the perceived level of subjectivity, vision is typically viewed as providing relatively *objective* information: Sweetser (1990) claims that vision is “our primary source of objective data about the world” (p.39)^3^. Along similar lines, Korsmeyer (1999) calls vision “phenomenally objective” (p.25). Not surprisingly, visual evidence is often considered as more reliable than auditory or other kinds of evidence [e.g., in grammaticalized evidentiality systems, Willett (1988), Aikhenvald (2004)]. In essence, we typically think of visual sensory experiences as eliciting relatively uniform sensory percepts across people.

In contrast to vision, the senses of taste and smell are typically regarded as conveying more *subjective* information and as involving more variable percepts across people (e.g., Viberg, 1984, 2001; Chafe and Nichols, 1986; Sweetser, 1990; Dubois, 2007). Evidence for a higher level of subjectivity for taste and smell relative to vision comes from both neurological data and lexical patterns. On the linguistic side, it has been observed that linguistic terms related to smell and taste (e.g., Buck, 1949; Krifka, 2010; Levinson and Majid, 2014; Winter, 2016) have a strong subjective component. Even the simple utterances “it's tasty” and “it's smelly” both convey subjective opinions [in opposite directions, see e.g., Krifka (2010)]. As Winter (2016) notes, there are many vision-based expressions that convey largely objective information (e.g., *striped, green*, and *round*), whereas words linked to taste and smell tend to be more subjective (e.g., *smelly, pungent*, and *delicious*).

Even after we abstract away from particular lexical items, non-linguistic neurological experiments provide evidence for the idea that sensations of taste and smell are inherently more linked to subjective evaluation than visual percepts. For example, the amygdala (linked to the olfactory bulb) exhibits increased blood flow for pleasant or unpleasant smells and tastes, but *not* for visual (or auditory) stimuli (e.g., Royet et al., 2000). As noted by Phillips and Heining (2002), neural evidence indicates that “emotion processing and perception of odors and flavors have similar neural bases” and that “olfactory and gustatory stimuli seem to be processed to a significant extent in terms of their emotional content, even if not presented in an emotional context” (p. 204). Broadly speaking, neural evidence indicates that smell and taste have similar neural bases and are more subjective than vision.

In sum, vision differs from taste and smell in terms of the perceived level of subjectivity: the visual modality is regarded as conveying more objective, non-opinion-dependent information, while taste and smell convey more subjective, opinion-based information. This core asymmetry between vision on the one hand, and taste and smell on the other, is summarized by Caballero and Paradis (2015) who note that “in contrast to the relatively objective and stable nature of visual elements in the world, the perceptions of smell, taste and touch are highly subjective and variable across human beings” (e.g., Viberg, 1984, 2001; Chafe and Nichols, 1986). Thus, in contrast to the visual domain (where Person A will tend to assume that she has roughly the same visual experience as Person B when they focus their visual attention on the same thing), in the domains of taste and smell A is less likely to assume that she has the same gustatory or olfactory experience as B when they eat or smell the same thing.

#### Access to the Relevant Perceptual Experience

In addition to differing levels of perceived subjectivity, vision, taste and smell differ in terms of the spatial relation that holds between the experiencer and the stimulus. This has consequences for who can have the relevant perceptual experience to be considered a potential attitude-holder, in a way that groups the modalities differently from what we saw in the preceding section.

Vision is traditionally viewed as a distal sense. Indeed, we can see things that are (relatively) far away, and no direct physical contact is needed between the visual stimulus and the perceiver. Early Greek philosophers already noted that with vision, “there is no evident contact between the perceived object and the organs of perception” (Korsmeyer, 1999, p.12). These characteristics mean that multiple people can easily experience the same visual stimulus. Indeed, unless our eyes are closed, sighted individuals are constantly exposed to on on-going stream of visual stimuli. As noted by San Roque et al. (2015), “As a distal sense, it seems likely that sight is one of the most readily and regularly shared perceptual experiences among interlocutors” (p. 50). They also note that visual cues are generally viewed as the basic foundation for joint attention (e.g., Moore and Dunham, 1995). In essence, in the domain of vision, multiple people can easily experience a visual stimulus and thus are potential attitude-holders for a PPT that describes the resulting percept.

In contrast to vision, taste is a proximal sense. To experience a gustatory stimulus, close contact—specifically, an event of something getting into one's mouth—is necessary. As noted by Elder et al. (2017, p. 878), “physical distance for taste is quite low because a stimulus must be within one's mouth in order to be sensed.” Although humans with normal vision are constantly experiencing visual stimuli during their waking hours, we typically only experience gustatory stimuli while eating. Thus, unlike vision, perceptual access to the relevant experience in the gustatory domain is limited to those who have a specific kind of proximate contact with the stimulus.

What about smell? The olfactory modality resembles vision in that—unlike with taste—no direct physical contact is needed between the stimulus and the experiencer, although greater physical proximity is required with smell than with vision: “[U]nlike with taste and touch, the stimulus can be sensed without any contact with the body. It simply must be close enough for the molecules to reach the nose” (Elder et al., 2017, p. 878). In sum, although access to olfactory experiences is not as unconstrainted as access to visual experiences, it is easier for multiple people to be potential attitude-holders for a PPT describing an olfactory experience as compared to a gustatory experience^4^.

Table 1 summarizes how these three sensory domains differ in (i) level of subjectivity and (ii) ease of access to the relevant perceptual experience.

**Table 1:** Vision, taste, and smell grouped by level of subjectivity and ease of perceptual access.

*Subjectivity*	vision < < smell, taste *less subjective more subjective*
*Access to the relevant perceptual experience*	vision < smell < < taste *less constrained access more constrained access*

As can be seen in Table 1, the olfactory modality has a mixed status in that it patterns like taste in terms of conveying subjective information, but is almost as unconstrained as vision in in terms of ease of perceptual access.

Linguistically speaking, the olfactory domain has been found to be unusual in another way as well: In many languages, the dedicated vocabulary for olfactory experiences is very limited (e.g., Yeshurun and Sobel, 2010) and people often struggle to name smells [but, importantly, this is not the case in all languages or cultures, Majid et al. (2018)]. Thus, while the sensory domains of vision and taste differ both in terms of perceived subjectivity and ease of perceptual access, the olfactory domain has a more mixed status, and is also linguistically less robustly encoded in English and many other languages. However, it is important to acknowledge that my experiments test U.S.-born native English speakers. Given crosslinguistic differences in the (in)effability of senses (e.g., Majid, 2021), the findings reported here should not be construed as linguistic universals, and further crosslinguistic experiments are needed.

### Identifying the Attitude-Holder of Subjective Adjectives

So far, we have focused on the differences between sensory modalities. Let us now take a closer look at a class of linguistic expressions, *predicates of personal taste* (PPTs), that can be used to express sensory experiences in different modalities and that have attracted extensive attention in theoretical semantics and philosophy [e.g., Lasersohn (2005), Stephenson (2007), Pearson (2013) and many others; see also Solt (2018) for recent experimental work].

Prior theoretical work on PPTs has approached them in a largely modality-neutral way. Potential differences between sensory modalities have received little if any direct attention. Researchers have mostly focused on sentences of the form “noun is adjective” (ex.1), where the sensory modality is not linguistically explicit. Sentences like (2), where the verb explicitly pins down the relevant sensory modality, have not been extensively or systematically discussed.

(1) The muffin was disgusting.
(2) The muffin looked/smelled/tasted disgusting.

Prior theoretical work on PPTs has tended to focus on the fundamental question of how to linguistically represent the fact that the meaning of these adjectives is judge-dependent—in other words, their interpretation is in some way relativized/anchored to the opinion or perspective of an attitude-holder. A number of different analyses have been proposed, but the present paper does not aim to distinguish between different formal accounts of judge dependence (e.g., Anand and Korotkova, forthcoming; Lasersohn, 2005; Glanzberg, 2007; Stephenson, 2007; Anand, 2009; Cappelen and Hawthorne, 2009; Patel-Grosz, 2012; Pearson, 2013; Snyder, 2013; Bylinina, 2014; Coppock, 2018; Rudin and Beltrama, 2019; Zakkou, 2019; Willer and Kennedy, 2020).

As regards the question of *attitude-holder identification* (when a comprehender encounters a PPT, how does s/he figure out whose opinion the PPT expresses?), prior work agrees that the first-person speaker is the default attitude-holder [aka the judge, e.g., Lasersohn (2005) on autocentric perspective].

#### Perspective-Shifting Away From the Default Attitude-Holder

Crucially, with PPTs, the first-person speaker is not the only possible attitude-holder [see e.g., Lasersohn (2005); for discussion regarding a range of perspective-sensitive elements see e.g., Karttunen and Zaenen (2005), Wang et al. (2005), Stephenson (2007), Amaral et al. (2008), Harris and Potts (2009), Kaiser (2015), Korotkova (2016), and Kaiser and Herron Lee (2017, 2018)]. For an example of shifting, let's imagine that ex. (3) is an excerpt from a novel. It has a first-person narrator, as indicated by the presence of “I” in the first clause. In addition, the excerpt mentions Eliza (whom I refer to as the “character”). In this context, given that Eliza is described as seeing the muffin, she could potentially also be construed as the attitude-holder in addition to (or instead of) the first-person narrator [For related experimental data, see Kaiser (2015), Kaiser and Herron Lee (2017, 2018)]. In other words, it seems possible in (3) to *shift away* from the default perspectival interpretation where the first-person narrator is the attitude-holder to an interpretation where the character is the attitude-holder (Note that in (3), *looked* is used in the final sentence, and both the narrator and the character can presumably see the muffin; I discuss this below).

(3) When I came into the room, Eliza saw the muffin on the platter. It looked disgusting.

The general question of perspective-shifting in fictional narrative contexts has been investigated by philosophers, linguists, literary narratologists and psychologists [see e.g., Banfield (1973), McHale (1978), Clark and Gerrig (1990), Fludernik (1993), Redeker (1996), Schlenker (2004), Lasersohn (2005), Sharvit (2008), Harris (2012), Eckardt (2015), Kaiser (2015), Maier (2015), Salem et al. (2015), Hinterwimmer (2017), Abrusán (2020), and many others; see also Bortolussi and Dixon (2003) and Klages et al. (2020) on perspective-shifting].

Crucially, prior work on narratives shows that not only is it possible to interpret the character as an attitude-holder of a PPT (such as *disgusting* in ex.3), but that subjective expressions like PPTs can in fact serve as *explicit cues to perspective-shift* from the narrator to the character: There exists a large body of narratological evidence indicating that subjective linguistic expressions in general act as cues to shift from the perspective of the narrator to the perspective of a character. Many of the more literary investigations did not look systematically at the specific class of PPTs, but they cite subjective adjectives such as *poor, dear, terrific, marvelous, awful*, and *stupendous* as examples of elements that are cues to perspective shift [see e.g., McHale (1978, p. 269) and Fludernik (1993, p. 26)]. More generally, in addition to subjective adjectives, other kinds of subjective expressions are also known to trigger perspective shift, including interjections and exclamations like *alas* and *oh*, expressions of uncertainty like *probably, perhaps*, as well as epithets like *that idiot, the jerk* and so on [e.g., McHale (1978), Fludernik (1993), see also Banfield (1973)].

These findings corroborate the intuition that in a narrative context like (3), the character can be construed as an attitude-holder of the PPT *disgusting*^5^. However, because prior work has not systematically investigated different sensory modalities, it is not yet known whether the likelihood of perspective shift (from narrator to character) is increased when a PPT is explicitly presented as involving a sensory experience, nor is it known whether the likelihood of perspective shift is modulated by the particular type of sensory modality.

#### Constraints on Identifying the Attitude-Holder

Even in narrative contexts, it is not the case that any character can freely be construed as the attitude-holder of a subjective adjective. There are constraints that guide this process. Of central relevance for the process of attitude-holder identification is the observation made in prior theoretical work that use of a PPT indicates that the attitude-holder must have *first-hand experience of the relevant kind* [see e.g., Pearson (2013), Ninan (2014) on the acquaintance inference, Bylinina (2014), Gunlogson and Carlson (2016), McNally and Stojanovic (2017), and Rudin and Beltrama (2019), inter alia]. In essence, for something to be judged fun or tasty, the person making this judgement must have the relevant experience. Usually, this someone is the default first-person speaker. For example, if I say (1), this suggests that I have the right kind of first-person experience (presumably direct gustatory, visual or olfactory experience; the prior theoretical semantics typically work does not make claims about specific modalities) on which to base my statement.

In other words, it has been claimed that PPTs entail that the attitude-holder is an *experiencer*, “a sentient individual who perceives the property in question” (McNally and Stojanovic, 2017, p. 24). The significance of the sentient experiencer is also discussed by Bylinina (2014): “A direct statement about someone's internal state can be made only if the judge parameter is set to the same value as the experiencer of this internal state” (Bylinina, 2014, p. 58). The attitude-holder/experiencer relation has been explored in depth, from a variety of perspectives, in several recent papers (e.g., Sæbø, 2009; Pearson, 2013; Ninan, 2014; Gunlogson and Carlson, 2016; Kennedy and Willer, 2016; Willer and Kennedy, 2020).

However, prior work in this tradition does not systematically distinguish between situations where an adjective is presented without a particular sensory modality (e.g., *It was disgusting*) vs. situations where an adjective is associated with a sensory modality (e.g., *It looked/smelled/tasted disgusting*). Nor does prior work in this vein make claims about differences between sensory domains. Thus, this raises the question: In narrative contexts, with sentences like (3) that have two possible attitude-holders, how does the requirement for the attitude-holder to have the relevant first-hand experience guide the process of attitude-holder identification when different sensory modalities are involved?

### Aims of the Present Work

The need for the attitude-holder to have the relevant kind of subjective, first-hand experience, coupled with the fact that sensory modalities differ in terms of their (i) level of subjectivity and (ii) ease of access to the relevant perceptual experience, suggests that sensory modality may guide the process of identifying the attitude-holder of subjective adjectives. However, despite the large body of work on the fundamental physiological and physical differences between sensory modalities (see the section entitled “Perceived Subjectivity”), this question has not been systematically addressed in prior linguistically-oriented research.

The question of whether and how the interpretation of PPTs depends on sensory modality has (i) implications for our understanding of perspective-taking—including whether information explicitly linked to a sensory modality is a stronger cue to perspective shift—as well as (ii) implications for theories of subjective adjectives. In existing semantic work, many analyses of PPTs' judge dependence seem to implicitly or explicitly center on the adjective itself. However, if we find that the attitude-holder of the *same* adjective [e.g., *disgusting* in (2)] can be interpreted differently depending on sensory modality, this suggests that accounts that treat attitude-holder identification as determined purely by the semantic properties of a specific class of adjectives are not sufficient. Instead, this kind of outcome would be more amenable to pragmatically-oriented theories of PPTs that allow for contextual, top-down effects to play a role, because such accounts could be extended to encompass differences between the senses even when the adjective itself is held constant.

To fill this empirical gap and to address the issues sketched out above, this paper reports three experiments that test whether sensory modality has an impact on how English-speaking comprehenders identify the attitude-holder of a subjective adjective in narrative contexts with two possible attitude-holders: a first-person narrator and a character mentioned in the story.

We consider three hypotheses about the effects of sensory modality on the process of attitude-holder identification. In the rest of this section, I introduce these hypotheses: the Sensory Experience Hypothesis, the Subjectivity-based Hypothesis and the Inference-based Hypothesis. The first focuses on the basic question of whether presenting a subjective adjective in a modality-neutral way vs. associated with a particular modality influences who is interpreted as the attitude-holder. The latter two make specific predictions regarding sensory modalities and are rooted in the differences reviewed in the sections entitled “Perceived Subjectivity” and “Access to the Relevant Perceptual Experience”.

To test these hypotheses, I used two-sentence sequences like ex.(4a-c), which describe a character having perceptual experiences in the visual, olfactory or gustatory domains respectively and present the critical PPT in predicative position using the matching sensory verb [See Anand and Korotkova (forthcoming) on attributive PPTs]. Thus, the sensory modality is expressed by means of the verb whose subject the character is (*Eliza saw/tasted/smelled*) and by means of the verb in the second sentence with the PPT (*it looked/smelled/tasted disgusting*). This ensures that it is clear to the reader that the character has a perceptual experience that “matches” the modality expressed in the PPT-containing sentence. I also compare these conditions to a baseline condition (ex.4d) where no perceptual experience is described and the PPT is presented with the verb *was*.

(4a) *Vision condition*
  When I came into the room, Eliza saw the muffin on the platter. It looked disgusting.
  Whose opinion is it that the muffin looked disgusting?
  The narrator's OR Eliza's
(4b) *Smell condition*
  When I came into the room, Eliza smelled the muffin on the platter. It smelled disgusting.
  Whose opinion is it that the muffin smelled disgusting?
  The narrator's OR Eliza's
(4c) *Taste condition*
  When I came into the room, Eliza tasted the muffin on the platter. It tasted disgusting.
  Whose opinion is it that the muffin tasted disgusting?
  The narrator's OR Eliza's
(4d) *Baseline*
  When I came into the room, Eliza put the muffin on the platter. It was disgusting.
  Whose opinion is it that the muffin was disgusting?
  The narrator's OR Eliza's

In ex.(4a), the vision condition, only Eliza is linguistically specified as seeing the muffin (she is the subject of the verb *see*), but both the narrator and Eliza are in the same room. Thus, we can infer they can both see the muffin^6^.

In ex.(4b), the smell condition, only Eliza is linguistically specified as smelling the muffin. However, again, given that she and the narrator are in the same room, both can be plausibly inferred as having the experience of smelling the muffin. However, the availability of the narrator as an attitude-holder is expected to be weaker than in the vision condition, because smell is more constrained by proximity than vision (see the section entitled “Access to the Relevant Perceptual Experience”).

In contrast, in ex.(4c), the taste condition, only Eliza is linguistically specified as tasting the muffin. Thus, comprehenders can infer that only Eliza has the relevant gustatory experience.

In ex.(4d), the baseline condition, Eliza is not linguistically described as seeing, tasting or smelling the muffin—she simply puts in on a plate.

In all conditions, the basic syntactic structures are the same. The only difference is the verb in the second clause (*Eliza saw/smelled/tasted/put*…) and the verb in the final sentence (*it looked/smelled/tasted/was disgusting*). After each text, participants were asked a question about the attitude-holder of the subjective adjective. The question used a verb that matches the sensory modality expressed in the preceding sentence. This allows us to test whether different sensory modalities influence the extent to which PPTs are able to trigger perspective-shifting away from the default attitude-holder—the first-person narrator—toward a character. Let us now turn to the predictions made by the three hypotheses about the likelihood of perspective-shift in each condition.

On a general level, it is possible that associating a subjective adjective with a specific sensory modality (regardless of what that modality is) will influence the process of attitude-holder identification, as compared to the same subjective adjective being presented in a context where no sensory modality is mentioned. I refer to this as the **Sensory Experience Hypothesis**. According to this hypothesis, describing the character as involved in *any kind* of sensory perception (e.g., *Eliza smelled/tasted/saw the muffin…*) makes perspective-shift to the character relatively more likely than in the baseline condition (e.g., *Eliza put the muffin…*). The predictions of this hypothesis regarding likelihood of perspective shift are in row (i) of Table 2 (Note that this hypothesis makes no predictions about differences *between* sensory modalities).

**Table 2:** Predictions of the three hypotheses.

	*Predicted likelihood of perspective shift to character's perspective (i.e., character is the attitude-holder)*
**General hypothesis:**	
Sensory Experience Hypothesis	(i) *baseline < < sensory experience*
**Additional hypotheses about differences between sensory modalities:**	
Subjectivity-based Hypothesis	(ii) *baseline < < vision < < {smell, taste}*
Inference-based Hypothesis	(iii) *baseline < < vision < smell < < taste*

More specific predictions about how different sensory modalities impact attitude-holder identification are made by the Subjectivity-based Hypothesis and the Inference-based Hypothesis.

According to the **Subjectivity-based Hypothesis**, the process of attitude-holder identification in contexts involving narrative fiction is guided by level of subjectivity (see the section entitled “Perceived Subjectivity”). Under this view, more subjective information acts as a stronger cue to shift from a narrator perspective to a character perspective than less subjective information. This prediction has its roots in the large body of research on perspective-shifting in narratives, which shows that *subjective linguistic expressions* function as a cue for readers to shift to the perspective of the character (see the section entitled “Perspective-Shifting Away From the Default Attitude-Holder”).

Given that the visual modality is more objective than taste and smell (as discussed in the section entitled “Perceived Subjectivity”), the Subjectivity-based Hypothesis predicts that in the smell and taste conditions (4b, 4c), comprehenders are more likely to perspective-shift from the narrator to the character—i.e., to interpret *disgusting* as conveying Eliza's opinion—as compared to the visual condition (4a). The baseline condition is predicted to trigger the lowest rate of perspective-shift. The predictions for the Subjectivity-based Hypothesis are in row (ii) of Table 2.

The predictions of the Subjectivity-based Hypothesis diverge from those of the **Inference-based Hypothesis**. This hypothesis posits that the process of attitude-holder identification is guided by comprehenders' inferences about who has access to the relevant perceptual experience. As discussed in the section entitled “Access to the Relevant Perceptual Experience”, (a) taste experiences require physical contact between the stimulus and the perceiver and thus are more constrained than either vision or smell, and (b) although neither smell nor vision require physical contact, smell is more constrained by proximity requirements than vision. If attitude-holder identification is shaped by inferences about who plausibly has access to the relevant sensory experience to be an attitude-holder, we expect that taste will clearly differ from both vision and smell, and that smell can also differ from vision.

In ex.(4c) with *taste*, Eliza is expected to be interpreted as the attitude-holder, as she is the only one with access to the relevant perceptual experience (tasting the muffin). Thus, taste should elicit the highest rate of perspective-shifts to the character. What about smell? Unlike the Subjectivity-based Hypothesis which groups taste and smell together, the Inference-based Hypothesis predicts that smell (ex.4b) patterns more like vision (ex.4a) than like taste, due to smell and vision having relatively less constrained perceptual access properties than taste. Smell is presumably somewhat more constrained than vision, though, given that it typically involves greater physical proximity to the stimulus. Thus, the Inference-based Hypothesis predicts more shifts from the narrator to the character's perspective with taste than with vision or smell, as well as more shifts with smell than with vision. The baseline condition is predicted to elicit the lowest number of perspective shifts to the character. These predictions are in row (iii) of Table 2 (The predictions of the three hypotheses regarding potential differences between sensory modalities are relative, not absolute).

The Inference-based Hypothesis has its roots in the general view that a central part of language processing has to do with context-sensitive inferences based on real-world knowledge that comprehenders make to arrive at a coherent interpretation of the discourse (e.g., Hobbs, 1979; Kehler, 2002). For example, Hobbs (1979) argues that pronoun interpretation is not governed by an independent mechanism (as many others have argued) but rather is a side-effect of comprehenders using real-world knowledge and reasoning to make inferences about how the components of a discourse fit together in a coherent way. Under the Hobbsian view, no special mechanism is needed for pronoun interpretation, beyond independently-needed reasoning and inferencing abilities that are rooted in our real-world knowledge. Thus, broadly speaking, the Inference-based Hypothesis can be viewed as a “cousin” of the coherence-based approach to pronoun interpretation. Thus, although the core assumption of the Inference-based Hypothesis (that we need to take seriously comprehenders' real-world inferences when considering aspects of language processing) has not previously been systematically tested in the domain of PPT interpretation, it is amply supported by prior work in other areas of language.

## Experiment 1

Experiment 1 tests whether and how information about sensory modality guides the process of attitude-holder identification. Experiment 1 used sequences like ex.(4a-d) and manipulated whether a character in the narrative has a perceptual experience in the visual, auditory or olfactory modality, to see whether this influences the likelihood of perspective shifting from the default first-person narrator to the character.

### Methods

#### Participants

Participants were recruited *via* Amazon Mechanical Turk and completed the experiment *via* the Qualtrics web interface. For all the studies reported in this paper, MTurk participants had to have a U.S. IP address, at least 1,000 previously-approved HITs and 98% or greater HIT approval rate. Participants received USD 1.50.

For Experiment 1, 56 native English speakers were included in the final data analysis. We only included those who self-reported being born in the U.S., speaking English as their first language (one person was excluded because of this), and having normal/corrected-to-normal vision and hearing (no one was excluded because of this) and who made no errors on four unambiguous catch trials (15 people excluded)^7^. In addition, three people were excluded to balance the number of participants per list.

In all experiments reported in this paper, exclusion criteria were pre-specified before data analyses on the target trials were conducted^8^. The research reported in this paper was reviewed and approved by the USC Institutional Review Board.

#### Design and Materials

Participants read two-sentence sequences (ex.4, repeated as ex.5), presented as extracts from novels, and answered questions about them. Each target started with a subordinate clause preamble that mentions the speaker/narrator by means of a first-person pronoun and describes the narrator as arriving at/entering the inferred location of the character. This clause is followed by a main clause that mentions a character by name. This set-up explicitly makes available two possible candidate attitude-holders (the narrator and the character e.g., Eliza, George, Amanda, Tim) for the “whose opinion” question that was presented after each target (Note that the question disambiguates “it” as referring to the muffin/relevant object, not the platter or something else). This question was presented on the same screen as the two-sentence sequence, to avoid a memory load, and was a two-alternative force-choice question. The answers provide a measure of who participants think is the attitude-holder of the PPT.

(5) When I came into the room, Eliza {saw/smelled/tasted/put} the muffin on the platter. It {looked/smelled/tasted/was} disgusting.
  Whose opinion is it that the muffin {looked/smelled/tasted/ was} disgusting?
  The narrator's OR Eliza's

The verbs were used to manipulate the senses involved in the item (vision, smell, taste, or no sense/baseline). Within an item, the PPT itself was kept constant in all conditions—this ensures that potential differences between the conditions cannot be attributed to the lexical semantics of particular adjectives. The adjectives were selected based on prior semantic work, and chosen so that they would be felicitous in the domains of smell, taste and vision (e.g., something can look, taste, or smell disgusting or amazing); both negatively-valenced and positively-valenced adjectives were included^9^. In each condition, the particular sensory domain was specified by the verbs in both the first and the last sentences, except for the baseline condition, where it was underspecified in both sentences. In the baseline condition, the verb *put* is used to describe the action done by the character, and the verb *was* is used in the second sentence.

The study included 24 target items, which used 12 different adjectives (specifically, predicates of personal taste; each used twice, see Appendix) and 24 different food items, as well as 42 filler items. The items were presented to participants in a Latin-Square design, so that no participant saw more than one version of each target. Variants where the preamble clause mentions the third-person character instead of the first-person narrator (e.g., *When she came into the room*…) were also included in the design, but are not reported here: They are not relevant for the perspective-shifting questions investigated in this paper because they do not explicitly introduce another potential attitude-holder.

#### Procedure

Participants were recruited from Amazon Mechanical Turk and completed the study at their own pace, over the internet. Participants read two-sentence sequences and answered questions about them (ex.5). The items were presented in writing. Each item was presented on a separate screen, but the critical sentences were displayed on the same screen as the multiple-choice question. Participants were told to imagine they were reading extracts from novels, and the term “narrator” was presented as part of the instructions.

#### Data Analysis

Data was analyzed using R (R Core Team, 2013). To compare the conditions to each other in order to assess effects of sensory modality on the process of attitude-holder identification, we fit logistic mixed effects regression models (glmer, lme4 1.1-20, Bates et al., 2015) to our data and used the emmeans package (emmeans 1.5.0, Lenth, 2018) to obtain Bonferroni-corrected pairwise comparisons. The proportion of “character's opinion” responses (1, 0) was used as the dependent variable; it is the inverse of the proportion of “narrator's opinion” responses. “Condition” was entered as a fixed effect into the model. As random effects, the models included random intercepts for subjects and items—as well as by-subject and by-item random slopes for the effect of condition when justified by model comparison—unless this resulted in singularity or non-convergence, in which case the model was further simplified [For each model, we started with the maximal random effect structure for subjects and items, and used model comparison to identify the maximal random effect structure justified by the design and supported by the data. Only random effects that contributed significantly to the model (*p* < 0.05) were included (Baayen et al., 2008)]. From-chance analyses were conducted using intercept-only logistic regression models.

### Results

**Figure 1:**
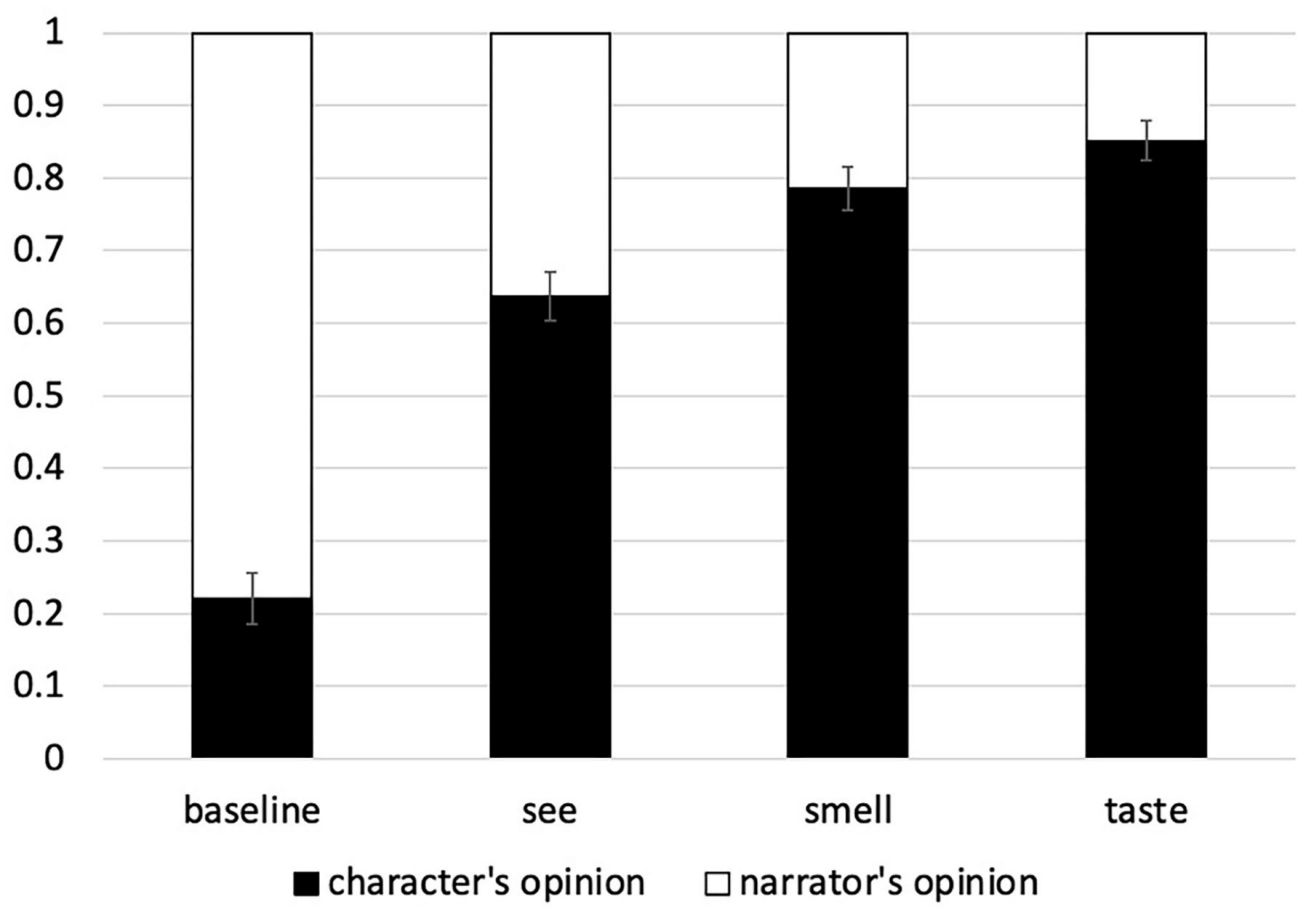
Proportion of character's opinion and narrator's opinion answer choices in Experiment 1. Error bars show +/−1 SE.

The proportion of “character's opinion” and “narrator's opinion” responses are shown in Figure 1. It's immediately clear that the baseline condition (no sensory modality specified) elicited mostly narrator responses and fewer than 25% character responses. This fits with the existing claims from the theoretical literature that the speaker (or writer) is the default attitude-holder of the PPT. Indeed, the proportion of character's opinion responses is significantly lower than chance (beta = −1.415, SE = 0.0298, *z* = −4.739, *p* < 0.0001).

The default preference to interpret the first-person narrator as the PPT attitude-holder vanishes in the other three conditions. Once the character in the narrative is described as the subject of a sensory verb (regardless whether it is seeing, smelling or tasting), that character becomes the preferred attitude-holder. Regardless of which sensory modality is specified, all three conditions elicit a higher-than chance rate of character responses (*taste*: beta = 2.881, SE = 0.764, *z* = 3.769, *p* < 0.001, *smell*: beta = 1.808, SE = 0.393, *z* = 4.601, *p* < 0.0001, *see*: beta = 0.8699, SE = 0.328, *z* = 2.653, *p* < 0.01).

When the conditions are compared directly to each other, the baseline condition elicits less character responses (and more narrator responses) than all of the other conditions (see Table 3 for statistical details). This supports the Sensory Experience Hypothesis.

**Table 3:** Pairwise comparisons for Experiment 1.

	**Estimate**	**SE**	***z*** **ratio**	***p***
Baseline—see	−2.482	0.311	−7.990	<0.0001
Baseline—smell	−3.487	0.350	−9.960	<0.0001
Baseline—taste	−4.080	0.385	−10.606	<0.0001
See—smell	−1.005	0.294	−3.422	0.0037
See—taste	−1.598	0.323	−4.943	<0.0001
Smell—taste	−0.0593	0.332	−1.790	0.4412

*P-value adjustment: Bonferroni method for six tests*.

*Shading indicates significance at p < 0.05*.

In addition, a closer look at the different sensory modality conditions shows that the rate of character responses is higher (and the rate of narrator lower) in the smell and taste conditions than the see condition (see Table 3). However, the taste and smell conditions do not differ significantly from each other (although smell elicits a numerically lower proportion of shifts to the character's perspective). Thus, although all three sensory conditions show a preference to interpret the character as the attitude-holder (rather than the narrator), this preference is stronger with taste and smell than with see, yielding the ranking *baseline* < *vision* < *{smell, taste}*, in line with the Subjectivity-based Hypothesis.

### Discussion

The results of Experiment 1 show that sensory modality has a significant impact on the process of identifying the attitude-holder of predicates of personal taste (PPTs). When no sensory modality is specified (baseline), the narrator is the preferred attitude-holder. As soon as a sensory modality is specified in the context, we see more shifts to the character's perspective. These findings support the Sensory Experience Hypothesis: The same PPT is interpreted differently depending on whether it is presented in a modality-neutral way (baseline) or explicitly associated with a sensory modality (the other three conditions).

We also find significant differences between the three sensory modalities: Contexts involving the gustatory and olfactory modalities elicit more shifts to the character's perspective than contexts involving the visual modality: *vision* < *{smell, taste}*. Even though smell is numerically in between vision and taste, statistically it does not differ from taste. This outcome fits best with the Subjectivity-based Hypothesis, according to which the process of attitude-holder identification is guided by the level of subjectivity associated with each sensory domain.

#### Potential Complication: On the Meanings of Smell

A potential concern with Experiment 1 arises from the polysemy of the transitive verb *smell* in English (and many other languages): When used in a transitive sentence, as in the clause *Eliza smelled the muffin*, the verb *to smell* can have an agentive interpretation (e.g., a person sniffs the muffin on purpose) or an experiencer interpretation (e.g., a person simply breathes the air and thereby becomes aware of a smell), e.g., Kopytko (1990), Gisborne (2010), and Dziwirek (2016). Gisborne (2010) describes two meanings in terms of the agent vs. experiencer distinction; similarly, Kopytko (1990) uses the labels [+active, + intent] and [-active, -intent] for these two meanings (*To taste* is semantically ambiguous in the same way, but in the contexts tested in this paper, the agentive interpretation is more salient. Thus, I focus here on *to smell*).

If the process of attitude-holder identification is guided by inferences about who has the relevant kind of experience (as posited by the Inference-based Hypothesis), these two meanings of the verb *smell* yield different predictions: If *smell* is interpreted as having the agentive meaning, the character is the most likely attitude-holder, as she is the one linguistically presented as the agent of the verb *smell*. In contrast, if *smell* has the experiencer meaning, both the character and the narrator are potential attitude-holders: Even the narrator, some distance away and not presented as the syntactic agent of smelling, can experience the smell.

If participants in Experiment 1 were interpreting the verb *smell* as having an agentive meaning, then—under the Inference-based Hypothesis—this would have boosted the character responses and made the smell condition pattern like the taste condition. This is indeed what we found. Thus, the conclusion that the results of Experiment 1 support the Subjectivity-based Hypothesis is too hasty, as the same outcome is also predicted by the Inference-based Hypothesis if the verb *to smell* is construed as having an agentive meaning^10^. So, rather than stemming from more subjective modalities triggering perspective shifting (in line with the Subjectivity-based Hypothesis), the results could instead be due to the polysemy of the verb *to smell* allowing for an interpretation that triggers an inference which favors the character as the attitude-holder (in line with the Inference-based Hypothesis).

## Experiment 2

Experiment 2 addresses the issue left open by Experiment 1 by making the experiencer meaning of the verb *to smell* more available. This was done by adjusting the stimuli to boost the availability of the narrator as an attitude-holder, by adding a speaker-oriented intensifier that modifies the PPT (e.g., *totally disgusting*) [see e.g., Athanasiadou (2007) and Rhee (2016)].

If the proportion of character responses in the smell condition of Experiment 1 was boosted to taste-like levels by the polysemy of the verb *smell* (in particular by its agentive meaning), the Inference-based Hypothesis predicts that once the narrator is made more available as an attitude-holder in Experiment 2, a difference will emerge between the taste and smell conditions. This is because once the narrator is boosted as a salient attitude-holder, the experiencer meaning of the verb *smell* (a person breathes and becomes aware of a smell) is also expected to become more available, given that the narrator's smelling can be inferred to be experiencer-oriented. In this case, we expect a lower rate of character opinion responses. Crucially, because this experiencer meaning is not available with the verb *taste* in the kinds of contexts we tested, the Inference-based Hypothesis predicts a difference between the taste and smell conditions in Experiment 2^11^.

To boost the availability of the narrator as an attitude-holder, Experiment 2 uses adverbial intensifiers (e.g., *totally, absolutely*). Adverbial intensifiers are an ideal tool for our aim of making the narrator's perspective more prominent, because a large body of work shows that they function as signs of the speaker's opinions/attitudes [e.g., Athanasiadou (2007), Waksler (2012), Rhee (2016), Beltrama (2018), see also Biber and Finegan (1988)]. For example, Beltrama notes that “the use of *totally* contributes to strengthening the speaker's commitment toward the utterance” (Beltrama, 2018, p. 119–220). More generally, Athanasiadou (2007) notes that these kinds of intensifiers “tend to be subjective in character and show involvement on the part of the speaker” (p. 560), and Rhee (2016) adds that this class “encodes evaluation or reflects the speaker's positionality” (p. 399). Based on this prior work, it is reasonable to expect that these intensifiers can make the first-person narrator more available as an attitude-holder.

Thus, in Experiment 2 adverbial intensifiers were added to the PPTs, with the goal of making the narrator's perspective more salient. In analyzing the results of this experiment, we first check whether the addition of the intensifier indeed had the predicted effect of making the narrator more available as an attitude-holder, and if so, whether this results in a difference emerging between the smell and taste conditions as predicted by the Inference-based Hypothesis.

### Method

#### Participants

Recruitment, payment and MTurk requirements were as in Experiment 1. Fifty-six new participants who had not done Experiment 1 were included in the final analysis. The exclusion criteria were the same as Experiments 1. In Experiment 2, three people were excluded for not being U.S.-born native English speakers and 20 for making errors on catch trials. Three additional people were excluded to balance the number of participants per list.

#### Design and Materials

The design was the same as Experiment 1, except that now, in all target items, the subjective adjective in the final clause was preceded by an intensifier (e.g., *totally, absolutely, really, extremely*), as shown in ex.(6).

(6) When I came into the room, Eliza {saw/smelled/tasted/put} the muffin on the platter. It {looked/smelled/tasted/was} really disgusting.
  Whose opinion is it that the muffin {looked/smelled/tasted/ was} really disgusting?
  The narrator's Eliza's

#### Procedure

The procedure was the same as Experiment 1.

### Predictions

If the narrator becomes more available as an attitude-holder due to the presence of intensifiers, then, according to the Inference-based Hypothesis, the experiencer-based meaning of the verb *to smell* should become more available and a difference between the smell and taste conditions should emerge. This is because experiencing a taste percept requires physical contact between the taster and the stimulus [something that only the character does in narratives like ex.(6)], whereas on the experiencer-based meaning of *to smell*, both the character and the narrator can experience the smell percept.

In contrast, the Subjectivity-based Hypothesis predicts that the results of Experiment 2 will pattern like Experiment 1, because the inherent (non-linguistic) level of subjectivity associated with the gustatory, olfactory, and visual modalities is not impacted by the addition of a speaker-oriented adverb.

### Results

**Figure 2:**
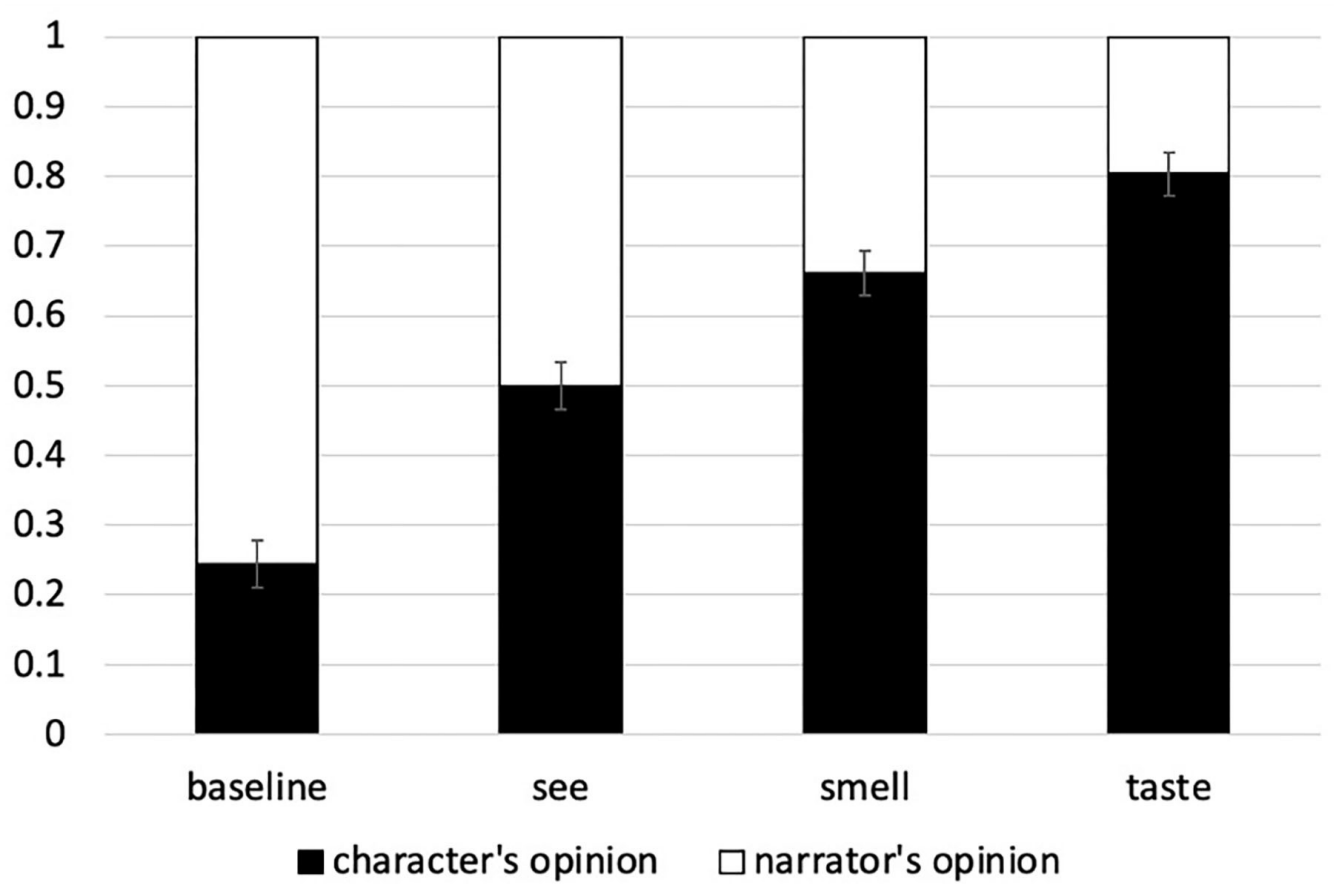
Proportion of character's opinion and narrator's opinion answer choices in Experiment 2.

The proportion of trials on which participants answered that the PPT reflects the opinion of the character is shown in Figure 2. As in Experiment 1, the proportion of narrator responses is the inverse of the character responses (due to the two-alternative forced-choice design). Like Experiment 1, in the baseline condition the proportion of character responses is significantly below chance (beta = −2.05, SE = 0.55, *z* = −3.73, *p* < 0.001), in contrast to the three sensory conditions: The proportion of character responses is significantly above chance in the taste and smell conditions (taste: beta = 2.235, SE = 0.553, *z* = 4.04, *p* < 0.0001), smell: beta = 1.122, SE = 0.382, *z* = 2.935, *p* < 0.001), and at chance in the see condition (beta = 0.016, SE = 0.33, *z* = 0.048, *p* > 0.96).

Before directly comparing the conditions to each other, let's first check whether the presence of intensifiers increases the availability of the narrator as an attitude-holder in the expected conditions, namely smell and see (i.e., contexts where the narrator could plausibly have access to the relevant experience). Indeed, the rate of narrator responses with smell and see is higher in Experiment 2 than Experiment 1 (*smell* Exp 2 vs. Exp 1: beta = 0.922, SD = 0.462, *z* = 1.996, *p* = 0.046; *see* Exp 2 vs. Exp 1: beta = 0.899, SD = 0.463, *z* = 1.941, *p* = 0.052). There are no significant differences between Experiments 1 and 2 in the baseline or the taste conditions, as expected (*p*'s > 0.3). The differences between Experiments 1 and 2 confirm that presence of an intensifier does indeed boost the likelihood of the first-person narrator being interpreted as the attitude-holder in exactly those contexts where the narrator can also be inferred to be a plausible experiencer.

Furthermore, when we compare the conditions to each other, we find clear differences between all four conditions. First, as in Experiment 1, the baseline condition elicits significantly fewer character responses than all three of the sensory experience conditions, in line with the Sensory Experience Hypothesis (statistical details are in Table 4). Second, as in Experiment 1, the taste and smell conditions elicit more character responses than the vision condition. However, now we also find significant differences between taste and smell: the rate of character responses is higher (and the rate of narrator responses lower) in the taste condition than in the smell condition. Thus, for the proportion of character's perspective interpretations, we find the following ranking: *baseline* < *see* < *smell* < *taste*. The distinction that now emerges between smell and taste is not predicted by the Subjectivity-based Hypothesis, but is compatible with the Inference-based Hypothesis.

**Table 4:** Pairwise comparisons for Experiment 2.

	**Estimate**	**SE**	***z*** **ratio**	***p***
Baseline—see	−1.695	0.305	−5.558	<0.0001
Baseline—smell	−2.688	0.329	−8.171	<0.0001
Baseline—taste	−3.731	0.370	−10.098	<0.0001
See—smell	−0.994	0.270	−3.564	0.0022
See—taste	−2.037	0.314	−6.493	<0.0001
Smell—taste	−1.043	0.304	−3.425	0.0037

*P-value adjustment: Bonferroni method for six tests*.

### Discussion

Experiment 2 set out to test a potential concern left open by Experiment 1, namely that the taste and smell conditions patterning together in terms of attitude-holder identification might be due *not* to similar levels of subjectivity (as proposed by the Subjectivity-based Hypothesis) but instead due to the polysemy of the verb *to smell* [agentive vs. experiencer meanings, e.g., Gisborne (2010)] boosting the rate of character responses, thereby making the smell condition look like the taste condition. Experiment 2 used adverbal intensifiers to boost the availability of the narrator as an attitude-holder, thus highlighting the experiencer meaning of the verb *to smell*, to see if this would reveal a difference between smell and taste conditions, as predicted by the Inference-Based Hypothesis.

Indeed, the results show that now, a significant difference between the smell and taste conditions emerges, exactly in the direction predicted by the Inference-based Hypothesis: The same subjective adjective is more likely to be interpreted as having the character as the attitude-holder in the taste condition than in the smell condition. According to the Inference-based Hypothesis, this is exactly what we expect, given that experiencing a taste percept requires the physical act of tasting [something that only the character does in narratives like ex.(6)] whereas we can infer that both the character and the narrator can experience the smell percept in these contexts. Crucially, the results of Experiment 2 are not predicted by the Subjectivity-based Hypothesis. According to this hypothesis, the outcome of Experiment 2 should have been just like Experiment 1, since the inherent (non-linguistic) level of subjectivity associated with the gustatory, olfactory and visual modalities does not change between experiments.

#### Multiple Potential Attitude-Holders

However, a potential concern with both Experiments 1 and 2 is that participants had to select a single attitude-holder when answering the forced-choice question about who is the attitude-holder (either the character or the narrator). There was no way to indicate an interpretation where *both* the narrator and the character share the opinion expressed by the subjective adjective. This is potentially problematic for the see and smell conditions, where it is possible for both the narrator and the character to have access to the relevant perceptual experience, and thus both could be inferred to be potential attitude-holders. Because Experiments 1 and 2 did not allow participants to report this kind of interpretation and forced them to opt for a binary response, one may wonder whether this constraint was distorting the results. To address this concern, in Experiment 3 participants had greater flexibility in indicating who they felt were the relevant attitude-holders: A third answer-choice was added that offers *both* the character and the narrator as possible attitude-holders. This allows us to assess whether the pattern predicted by the Inference-based Hypothesis—specifically the claim that vision and smell pattern more alike than taste—arises even when participants are free to select both the character and the narrator as attitude-holders.

## Experiment 3

### Methods

#### Participants

Recruitment, payment and MTurk requirements were as in Experiments 1 and 2. Sixty-four new participants who had not done Experiment 1 or 2 were included in the final analysis. The exclusion criteria were the same as above. Four people were excluded for not being U.S.-born native English speakers, one for reporting a hearing impairment and 26 for making errors on catch trials.

#### Design and Materials

The design and materials were the same as Experiments 1 and 2, except that now, the multiple choice question after each item had three answer choices (ex.7): Participants now had the additional option of selecting *both* the narrator and character as attitude-holders.

(7) When I came into the room, Eliza {saw/smelled/tasted/put} the muffin on the platter. It {looked/smelled/tasted/ was} disgusting.
  Whose opinion is it that the muffin {looked/smelled/tasted/ was} disgusting?
  The narrator's
  Eliza's
  Both the narrator and Eliza have this opinion.

#### Procedure

The procedure was the same as Experiments 1 and 2.

#### Data Analysis

Data analyses were conducted in the same way as in Experiments 1 and 2, except that—since the dependent variable now has three levels—we conducted three analyses: One analysis on the proportion of *character's opinion* responses (with character's opinion responses coded as 1 and all other responses as 0), a second on the proportion of *narrator's opinion* responses (with narrator's opinion responses coded as 1 and all other responses as 0), and a third on the proportion of *both* responses (with both responses coded as 1 and all other responses as 0).

### Predictions

The broad predictions about perspective-shift are the same as for Experiments 1 and 2: The Subjectivity-based Hypothesis predicts that the taste and smell conditions will pattern alike, differently from vision. In contrast, the Inference-based Hypothesis predicts that taste and smell will differ, with smell falling in-between taste and vision.

### Results

**Figure 3:**
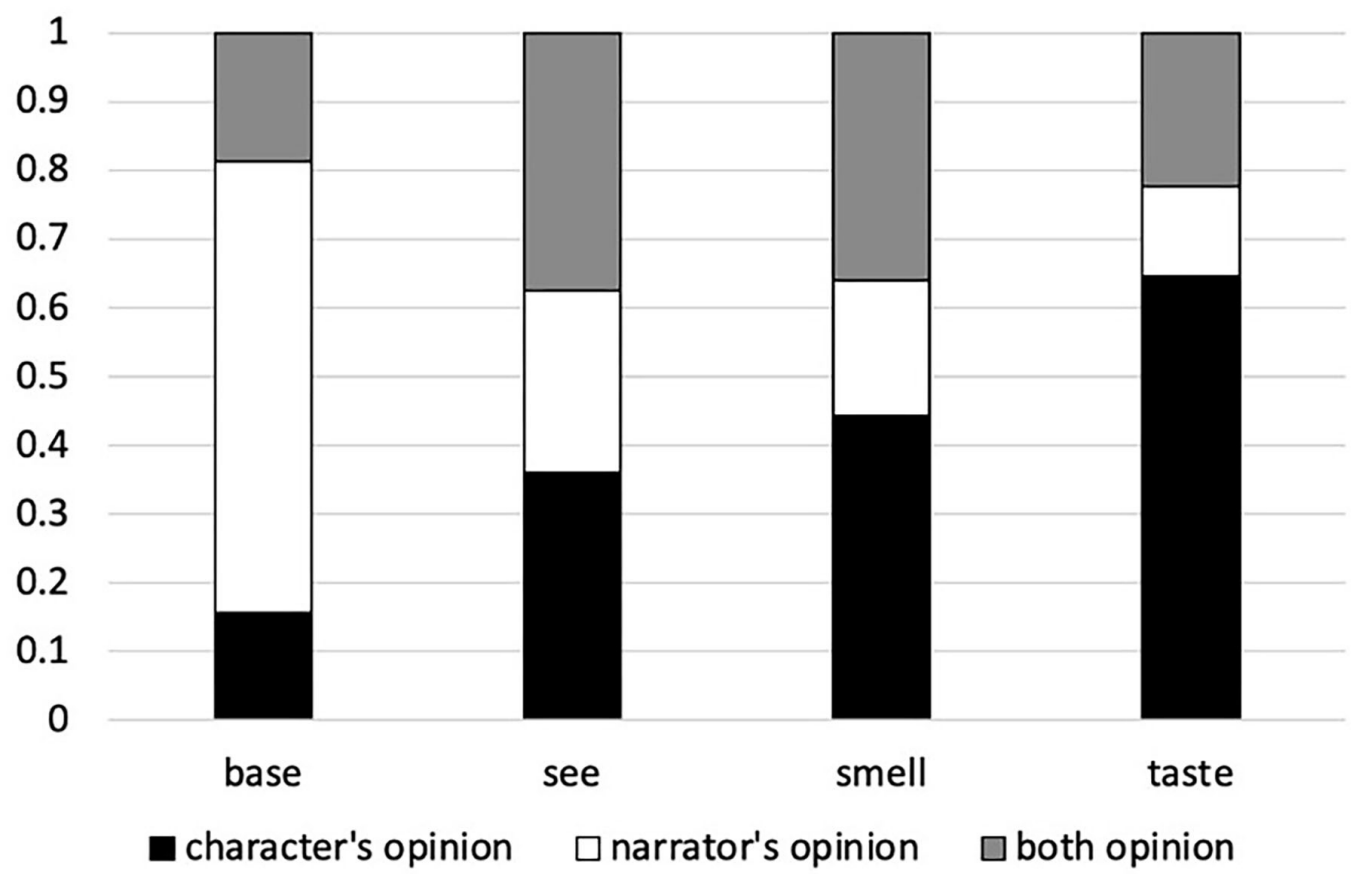
Proportion of character's opinion, narrator's opinion and both answer choices in Experiment 3.

Figure 3 shows the proportion of character's opinion, narrator's opinion and both opinion responses for each of the four conditions. The outcomes of the statistical analyses are reported in Tables 5A–**C**. As in Experiments 1 and 2, the baseline condition elicits the most narrator responses: When no sensory modality is specified, participants are most likely to interpret the narrator as the attitude-holder of the subjective adjective. Indeed, the proportion of narrator responses is significantly higher in the baseline condition than in the other three conditions (Table 5B). This fits with the Sensory Experience Hypothesis. Next, we consider the differences between the three sensory modalities.

**TABLE 5A:** Character's opinion responses: pairwise comparisons for Experiment 3.

	**Estimate**	**SE**	***z*** **ratio**	***p***
Baseline—see	−1.868	0.339	−5.505	<0.0001
Baseline—smell	−2.511	0.349	−7.194	<0.0001
Baseline—taste	−4.166	0.406	−10.251	<0.0001
See—smell	−0.643	0.288	−2.233	0.1533
See—taste	−2.297	0.332	−6.921	<0.0001
Smell—taste	−1.654	0.317	−5.216	<0.0001

*P-value adjustment: Bonferroni method for six tests*.

**TABLE 5B:** Narrator's opinion responses: pairwise comparisons for Experiment 3.

	**Estimate**	**SE**	***z*** **ratio**	***p***
Baseline—see	2.496	0.298	8.377	<0.0001
Baseline—smell	3.075	0.325	9.468	<0.0001
Baseline—taste	3.827	0.371	10.307	<0.0001
See—smell	0.582	0.303	1.923	0.3269
See—taste	1.331	0.340	3.919	0.0005
Smell—taste	0.749	0.347	2.162	0.1836

*P-value adjustment: Bonferroni method for six tests*.

#### See vs. Taste

There are more narrator responses and both responses with see than taste (Tables 5B,C) and more character responses with taste than see (Table 5A)—echoing the high rate of perspective-shifts to the character with taste that we saw in Experiments 1 and 2. This difference is predicted by both the Subjectivity-based Hypothesis and the Inference-based Hypothesis.

**TABLE 5C:** Both (character and narrator's) opinion responses: pairwise comparisons for Experiment 3.

	**Estimate**	**SE**	***z*** **ratio**	***p***
Baseline—see	−1.409	0.286	−4.920	<0.0001
Baseline—smell	−1.306	0.286	−4.565	<0.0001
Baseline—taste	−0.319	0.295	−1.079	1.000
See—smell	0.103	0.254	0.404	1.000
See—taste	1.090	0.275	3.967	0.0004
Smell—taste	0.988	0.275	3.593	0.0020

*P-value adjustment: Bonferroni method for six tests*.

#### Smell vs. Taste

The smell condition elicits fewer character responses, more both responses and numerically more narrator responses^12^ than the taste condition (Tables 5A–C). Broadly speaking, as in Experiment 2, taste elicits more perspective-shifts to the character than smell. Thus, these two conditions do not pattern alike, contrary to the predictions of the Subjectivity-based Hypothesis and in line with the Inference-based Hypothesis.

#### Smell vs. See

Numerically, the smell condition elicits more character responses (smell: 44.27% vs. see: 35.94%), fewer narrator interpretations (smell: 19.79% vs. see: 26.56%)^13^ and comparable numbers of both responses as the see condition (35.94% vs. 37.5%). Although these differences do not reach significance, they resemble the finding in Experiment 2 that the smell condition is in-between the see and the taste conditions in terms of the likelihood of perspective-shifts to the character.

The comparable numbers of both responses fit with the inference-based view that the character and the narrator are both plausible attitude-holders in the smell and see conditions. In line with this line of thinking, the proportion of both responses is significantly higher in the see and smell conditions than in the taste or the baseline condition (Table 5C)—which is what the Inference-based Hypothesis leads us to expect.

### Discussion

Experiment 3 set out to address a potential concern with Experiments 1 and 2, namely that by forcing participants to choose only one attitude-holder, we may inadvertently have distorted the data. Thus, in Experiment 3 three answer choices were provided, so that participants could select the narrator, the character or *both* as attitude-holders.

As a whole, the results of Experiment 3 are more in line with the Inference-based Hypothesis—as well as the general Sensory Experience Hypothesis—than with the Subjectivity-based Hypothesis: In a flexible situation where participants are able to select both the character and the narrator as potential attitude-holders, we replicate the earlier differences between the baseline condition vs. all three sensory conditions (predicted by the Sensory Experience Hypothesis) and the differences between smell and taste (in line with the Inference-based Hypothesis and contrary to the predictions of the Subjectivity-based Hypothesis). Moreover, the gradient numerical differences between see, smell and taste (in order of increasing perspective-shift to the character) and the high numbers of both responses with see and smell fit best with the Inference-based Hypothesis.

## General Discussion

The interpretation of subjective adjectives such as *amazing, disgusting, interesting*, and *enticing* depends on an attitude-holder with the relevant kind of experience. To judge, say, a cake as tasting or smelling amazing, the attitude-holder must have tasted or smelled the cake. However, although the importance of the relevant kind of first-person experience has been acknowledged in prior work on attitude-holder identification, potential differences between sensory modalities have not been systematically investigated. The present work investigates whether and how the process of identifying the attitude-holder is influenced by the sensory modality of the subjective experience. Short narratives were used to test if sensory modality has an effect on whether a predicate of personal taste (PPT) is interpreted as reflecting the point-of-view of the default attitude-holder (first-person narrator), or shifted to the point-of-view of a character in the narrative. The experiments manipulated whether the PPT referred to a visual, olfactory or gustatory experience (e.g., *Eliza smelled/tasted/saw the muffin … The muffin looked/smelled/tasted disgusting*) or was presented in a modality neutral way (e.g., *Eliza put the muffin … The muffin was disgusting*).

### Perspectival Consequences of Being Linguistically Realized as a Sensory Experiencer

Let us first consider the Sensory Experience Hypothesis. According to this hypothesis, presenting the character as the subject of any kind of sensory perception verb (e.g., *Eliza smelled/tasted/saw the muffin…*) makes a perspective-shift from the narrator to the character more likely than if the character is not presented as a sensory experiencer (e.g., *Eliza put the muffin…*). Indeed, all three experiments support this hypothesis: Mention of any sensory modality—whether it be visual, olfactory or gustatory—triggers more perspective-shifting than the baseline condition where no sensory experience is mentioned. Though this finding does not contradict current semantic theories of subjective adjectives, it is not directly predicted by them. As a whole, the finding that mention of any sensory modality increases the likelihood of perspective-shift highlights the importance of contextual factors and the role that pragmatic inferences play in the interpretation of PPTs.

Because the same PPTs were used in all version of an item, these results cannot be attributed to the lexical semantics of particular adjectives. By using the same adjective in all versions of an item, the present work differs from most prior linguistic work on sensory vocabulary which has tended to focus on specific lexical items related to different senses (e.g., *pungent* for smell, *delicious* for taste). Using the same subjective adjectives in all four conditions (e.g., *disgusting, amazing*), the present work shows that the effect of sensory domain is not restricted to the lexical semantics of certain adjectives. Furthermore, this approach has the advantage of not requiring us to identify certain adjectives as being linked to a certain sensory modality. As Winter (2016) notes, sensory terms are often multimodal (p. 976), and assigning sensory modalities to adjectives is non-trivial. In the present work, I sidestep this concern by using the verbs to unambiguously indicate what the modality is.

### Differences Between Sensory Modalities

Having established that explicit presentation as a sensory experiencer boosts the likelihood of being identified as the attitude-holder, we can now ask whether there are further differences between the sensory domains of taste, smell and vision. According to the Subjectivity-based Hypothesis, more subjective information acts as a stronger cue to shift to the character's perspective than less subjective information. Since the visual modality is regarded as conveying more objective information than taste or smell (see the section entitled “Perceived Subjectivity”), the Subjectivity-based Hypothesis predicts that comprehenders are more likely to perspective-shift from the narrator to the character with taste and smell than with vision (*vision* < *smell, taste*). In contrast, the Inference-based Hypothesis posits that the process of attitude-holder identification is guided by comprehenders' inferences about who has access to the relevant perceptual experience. Based on differences between modalities regarding the need (or lack thereof) of physical contact and proximity to the stimulus (see the section entitled “Access to the Relevant Perceptual Experience”), this hypothesis predicts that perspective-shift is more likely with taste than smell or vision (physical contact vs. no physical contact needed). This hypothesis is also compatible with more perspective-shifts with smell than vision: Although neither smell nor vision require physical contact, perceiving smells is more governed by physical proximity than perceiving visual input (*vision* < *smell* < < *taste*).

Furthermore, because the Inference-based Hypothesis derives the differences between conditions from comprehenders' inferences about who is most likely to have the relevant experience, contextual factors that modulate these inferences can also play a role. The Subjectivity-based hypothesis does not predict sensitivity to contextual factors, because the inherent (non-linguistic) subjectivity of the different sensory modalities stems from their biological and neural properties, which do not change in different linguistic contexts.

Put together, the results of the three experiments presented here provide more support for the Inference-based Hypothesis than for the Subjectivity-based Hypothesis. The taste condition triggers more perspective-shifting to the character than the vision condition in all three studies, as predicted by both hypotheses. At first glance, the results of Experiment 1 appear to show attitude-holder identification in the smell condition patterning like the taste condition, but Experiment 2 shows that once we address the polysemy associated with the agentive and experiencer meanings of the verb *smell*, the smell condition no longer patterns like the taste condition. Experiment 3 corroborates the result from Experiment 2 by showing that even if participants have the possibility of selecting multiple attitude holders, the smell condition diverges from the taste condition. These findings support the Inference-based Hypothesis for English. Overall, there is no convincing evidence of smell and taste consistently patterning more alike than smell and vision, contrary to the predictions of the Subjectivity-based Hypothesis.

The conclusion that inferences about who has access to the relevant sensory experience guide the process of attitude-holder identification fits with the general view that much of language processing has to do with inferences based on real-world knowledge that comprehenders make to arrive at a coherent interpretation of the discourse [Hobbs (1979), see also Kehler (2002)]^14^. This coherence-based approach captures many key aspects of pronoun use and interpretation and is supported by a growing body of pronoun-focused research [see e.g., Kehler and Rohde (2013)]. Although the present paper does not argue for a direct equivalence between pronoun interpretation and attitude-holder identification, it is (to the best of my knowledge) the first to provide experimental evidence that the process of attitude-holder identification with subjective predicates is sensitive to inferences rooted in the real-world differences between sensory modalities coupled with information from the linguistic context.

#### On the Variability of Smell

Compared to the visual and gustatory condition, the results for the olfactory conditions in all three experiments in this paper are relatively more variable. While this may at first glance seem unexpected, there are multiple reasons why the process of attitude-holder identification in the smell condition can be more variable than with vision or taste. First, on a non-linguistic level, the sensory modality of smell is in-between taste and vision in terms of not requiring direct physical contact but still requiring some level of physical proximity (as discussed in the section entitled “Access to the Relevant Perceptual Experience”), the level of which could depend on the strength and type of the smell as well as other contextual factors. This is likely to make inferences about potential attitude-holders more variable with smell than with vision or taste. Another potential source of variation is the fact that, on the linguistic level, in English the verb *to smell* is ambiguous between an agentive, intentional act of smelling vs. a non-agentive, non-volitional act of experiencing a smell (see the section entitled “Potential Complication: On the Meanings of Smell”). These two shades of meaning add further variation to the inferences that comprehenders can draw, as we already saw in the differences between Experiments 1 and 2. Third, it is possible that inferences about smell being less stable than vision or taste stems from the same (not-yet-fully-understood) reason that underlies the impoverished nature of the lexicon for smell in English and many other languages and people's struggles with naming smells [see the section entitled “Access to the Relevant Perceptual Experience”, e.g., Yeshurun and Sobel (2010)]. Future research on languages with richer lexicons for smell would shed light on this.

Crucially, this kind of variability between the smell conditions and the other conditions is fully compatible with the Inference-based Hypothesis, as this hypothesis allows for multiple factors to influence the kinds of inferences made by comprehenders—in fact, this is a key prediction made by the Inference-based Hypothesis. This contrasts with the Subjectivity-based Hypothesis, under which the level of subjectivity attributed to each modality is fixed, and thus the process of attitude-holder identification is not expected to change based on contextual factors.

### Implications for Semantic Theories of Predicates of Personal Taste (PPTs)

Experiments 1, 2, and 3 show that the same subjective adjective can be interpreted differently depending on whether it is presented in a modality-neutral way (baseline condition) or explicitly associated with a sensory modality (the other three conditions). These results pose challenges for analyses of judge dependence that are purely centered on the adjective, as they suggest that accounts that only focus on the adjective itself and do not take the context (including the rest of the sentence) into account are not sufficient.

Many prior semantic accounts of PPTs seem to be largely focused on the semantic representation of the adjective itself [e.g., Lasersohn's (2005) judge-based analysis] and thus do not appear to straightforwardly predict the results reported here. Although these accounts are not incompatible with my results, if one wants to maintain a purely semantic approach, it seems that some kind of additional mechanism—perhaps one that is able to operate directly on the assignment of the judge variable/parameter, if we are following an approach that uses such a variable or parameter—would be needed to derive the differences found in Experiments 1, 2, and 3. Under a view where the lexical entries of PPTs have a special structure involving a judge parameter (e.g., Lasersohn, 2005; Bylinina, 2014), it seems that (under a purely semantic approach) to capture the results reported in this paper one might need to complicate the lexical entries of these adjectives.

The results reported in the present paper fit more easily with approaches to PPTs that seem more amenable to pragmatic, top-down effects stemming from the differences between the senses. One such approach has been proposed by Kennedy and Willer (2016), who did not look at sensory modalities but who make the point, more generally, that subjectivity is a highly context-sensitive, pragmatic phenomenon that “is not to be explained strictly in terms of any particular semantic parameter, implicit argument, or lexical underspecification” (p. 292). In more recent work, Willer and Kennedy (2020) suggest that experiential attitudes (resulting from the experiences that allow people to make judgements about taste and other subjective matters) can ultimately be captured in terms of a “functionalist analysis,” such that “they are to be characterized in terms of the role they play in the *cognitive system* of which they are a part and, specifically, in terms of *their causal relations to sensory stimulations*, other mental states, and behavior” (p. 9, italics added). Thus, Willer and Kennedy acknowledge the need for a linguistic account of subjective expressions to interface with sensory experiences and a broader cognitive system. Moreover, in a different line of research, Rudin and Beltrama (2019, p. 96–99) sketch out an approach that argues against subjective predicates having special lexical semantics and instead emphasizes the role of world knowledge and different kinds of inferences. It seems that these kinds of approaches are, at least on a broad level, compatible with the results reported here regarding the differences between sensory modalities, which I suggest are best captured by the Inference-based Hypothesis.

## Data Availability Statement

The raw data supporting the conclusions of this article will be made available by the authors, without undue reservation.

## Ethics Statement

The studies involving human participants were reviewed and approved by the University of Southern California Institutional Review Board. Written informed consent for participation was not required for this study in accordance with the national legislation and the institutional requirements.

## Author Contributions

The author confirms being the sole contributor of this work and has approved it for publication.

## Conflict of Interest

The author declares that the research was conducted in the absence of any commercial or financial relationships that could be construed as a potential conflict of interest.

## Publisher's Note

All claims expressed in this article are solely those of the authors and do not necessarily represent those of their affiliated organizations, or those of the publisher, the editors and the reviewers. Any product that may be evaluated in this article, or claim that may be made by its manufacturer, is not guaranteed or endorsed by the publisher.

## Footnotes

^1^I use the term “attitude-holder” as a non-theoretical, descriptive term for the individual whose perspective/attitude is reflected by the subjective adjective or other subjective element. I set aside generic readings, where the attitude-holder is conceptualized as “people in general” [see e.g., Moltmann (2010), Pearson (2013) for discussion], as they are not salient in the contexts investigated here.

^2^I do not investigate hearing, touch, proprioception, or skin senses (e.g., temperature). This is due to challenges associated with incorporating them into the experiment: As explained below, items in the different conditions only differed with respect to the modality-indicating verb. All other aspects, including adjectives, were held constant. This ensures that any differences between modalities are not due to specific adjectives. Given these constraints, it was not possible to create plausible items involving more than three senses while also using the semantically relevant kind of adjectives.

^3^The link between vision and objective information has been claimed to explain why, in many Indo-European languages, verbs of vision develop cognition-related meanings (e.g., Sweetser, 1990). However, there are Australian languages where verbs of *audition* develop such meanings (e.g., Evans and Wilkins, 2000). Thus, there is crosslinguistic and cross-cultural variation in this regard.

^4^This section has a non-linguistic point-of-view—it is about sensory perception *independent* of linguistic encoding. In the linguistic domain, sensory verbs differ in terms of agentivity and intentionality/volition. E.g., *to look* conveys an agentive, intentional action; *to see* does not. In terms of their linguistic argument structure, *to smell* and *to taste* allow for both an agentive and non-agentive/experiencer-type interpretation (e.g., Gisborne, 2010). I discuss this more in the section entitled “Potential Complication: On the Meanings of Smell”.

^5^There exists a large literature on different kinds of perspective-shifting phenomena in language, including free indirect discourse [free indirect speech, e.g., Sharvit (2008), Eckardt (2015)], protagonist projection (e.g., Abrusán, 2020), viewpoint shift [e.g., Hinterwimmer (2017); see also Hinterwimmer (2019)], psychonarration and narrated perception [e.g., Fludernik (1993), p. 311 for an overview]. The present work does not distinguish these potentially overlapping phenomena, as the differences between them are not relevant for the central aims of this paper.

^6^The first sentence (*Eliza saw the muffin*) uses *to see* instead of *to look*, to make the argument structural properties of the perception verbs (*saw/smelled/tasted*) as comparable as possible. The lexical semantics of the three verbs in the experiments all allow for an experiencer-based interpretation, unlike *look at* which is hard-wired for only an agentive reading (e.g., Kopytko, 1990; Gisborne, 2010) and deserves to be investigated in future work. Thus, the verbs in the current studies are unified in not requiring their subjects to be intentional agents. I discuss the verbs more in the section on “Potential Complication: On the Meanings of Smell”.

^7^We asked about (non-correctable) vision and hearing impairments, because auditory and visual impairments can limit individuals' access to English-language input. The catch trials were two-sentence sequences like the targets, but did not describe sensory experiences. All four catch trials were followed by a forced-choice question that had a clear correct answer, For example, “Sandy heard that Destiny and Julie are cruel to their pets. Destiny dragged the kitten with the string” was followed by the question “Who had the string?” and the answer choices were “Destiny” and “Sandy”.

^8^A potential concern with MTurk is the decrease in data quality that researchers have recently observed (e.g., Chmielewski and Kucker, 2020; Kennedy et al., 2020). Given these concerns, we used catch trials to exclude participants, in line with the advice of Chmielewski and Kucker. Our exclusion numbers may seem high, but are in line with or lower than Chmielewski and Kucker's: They found 38–62% of MTurk participants failing at least one data quality validity indicator in summer/fall 2018 and spring 2019.

^9^Given that these studies focus on PPT adjectives (a semantically restricted set), it was not possible to specifically match for levels of sensorimotor strength, but crucially all adjectives could be felicitously used with the three sensory domains.

^10^Using *sniff* instead of *smell* does not solve this concern, as *sniff* is typically interpreted as only having the agentive meaning.

^11^These predictions could be recast in terms of agentivity, in a way that I regard as compatible with the Inference-based Hypothesis: If we make the agentive meaning of the verb *to smell* less available—in a context where the verb *to taste* only has an agentive meaning—we predict a decrease in perspective-shifts to the character's viewpoint. I regard an agentivity-based account as a sub-case of the inference-based account, because the effects of agentivity can be plausibly attributed to comprehenders' inferences being sensitive to who is linguistically presented as the agent of a particular verb.

^12^The narrator responses do not differ significantly after Bonferroni correction (Table 5B), but before Bonferroni correction, the smell condition elicits more narrator responses than the taste condition (*p* = 0.03).

^13^The differences in the proportion of character responses and narrator responses between the see and the smell conditions are not significant after strict Bonferroni correction (see Tables 5A,B), but they are significant or marginal without Bonferroni (smell vs. vision character responses *p* = 0.03, narrator responses *p* = 0.054).

^14^Future work on the Inference-based Hypothesis could use sensorimotor norms (e.g., Lynott and Connell, 2009; Lynott et al., 2020) to investigate how fine-grained ratings about the sensory properties of subjective adjectives modulate the process of attitude-holder identification.

## APPENDIX

The target stimuli used in the experiments. The adverbial intensifiers shown in parentheses were only used in Experiment 2.

1. Right after I entered the room, Emily {saw/smelled/tasted/put} the salad in the bowl. It {looked/smelled/tasted/was} (extremely) revolting.

2. After I entered the room, Tim {saw/smelled/tasted/put} the cookie on the shelf. It {looked/smelled/tasted/was} (really) dreadful.

3. When I got home, Joe {saw/smelled/tasted/put} the fish in the pan. It {looked/smelled/tasted/was} (absolutely) horrifying.

4. When I entered the room, Jane {saw/smelled/tasted/put} the pretzel in the bowl. It {looked/smelled/tasted/was} (truly) dreadful.

5. As soon as I stepped into the room, Alex {saw/smelled/tasted/put} the baked potato on the counter. It {looked/smelled/tasted/was} (truly) delicious.

6. Right after I walked in, Lisa {saw/smelled/tasted/put} the cake on the table. It {looked/smelled/tasted/was} (absolutely) amazing.

7. Right after I arrived, Tina {saw/smelled/tasted/put} the baguette on the rack. It {looked/smelled/tasted/was} (really) interesting.

8. As soon as I walked in, Luke {saw/smelled/tasted/put} the donut in the basket. It {looked/smelled/tasted/was} (extremely) pleasant.

9. After I arrived at the party, Kevin {saw/smelled/tasted/put} the cupcake in the box. It {looked/smelled/tasted/was} (incredibly) enticing.

10. As soon as I arrived, Charlotte {saw/smelled/tasted/put} the pancake on the plate. It {looked/smelled/tasted/was} (totally) delightful.

11. As soon as I came home, Amanda {saw/smelled/tasted/put} the quesadilla in the skillet. It {looked/smelled/tasted/was} (truly) amazing.

12. As soon as I went into the room, Chris {saw/smelled/tasted/put} the cookie in the jar. It {looked/smelled/tasted/was} (extremely) interesting.

13. When I came into the room, Eliza {saw/smelled/tasted/put} the muffin on the platter. It {looked/smelled/tasted/was} (really) disgusting.

14. After I got home, Billy {saw/smelled/tasted/put} the hamburger on the tin foil. It {looked/smelled/tasted/was} (extremely) unpleasant.

15. When I returned home after work, George {saw/smelled/tasted/put} the pork in the pot. It {looked/smelled/tasted/was} (totally) horrifying.

16. When I arrived, Sarah {saw/smelled/tasted/put} the ice cream in the bowl. It {looked/smelled/tasted/was} (really) tantalizing.

17. After I got home from school, Michael {saw/smelled/tasted/put} the stir-fry in the dish. It {looked/smelled/tasted/was} (very) pleasant.

18. As soon as I came to the party, Todd {saw/smelled/tasted/put} the biscuit on the stove. It {looked/smelled/tasted/was} (absolutely) delightful.

19. As soon as I walked into the room, Mary {saw/smelled/tasted/put} the pizza in the tupperware. It {looked/smelled/tasted/was} (very) tantalizing.

20. When I arrived at the gathering, Ted {saw/smelled/tasted/put} the hot dog on the tray. It {looked/smelled/tasted/was} (really) enticing.

21. When I returned to the living room, Michelle {saw/smelled/tasted/put} the popcorn in the container. It {looked/smelled/tasted/was} (really) delicious.

22. After I entered the office, Bob {saw/smelled/tasted/put} the bagel on the counter. It {looked/smelled/tasted/was} (truly) disgusting.

23. Right after I arrived at the tea party, Linda {saw/smelled/tasted/put} the brownie on the baking sheet. It {looked/smelled/tasted/was} (absolutely) revolting.

24. Right after I entered, Denise {saw/smelled/tasted/put} the pasta in the pot. It {looked/smelled/tasted/was} (incredibly) unpleasant.
